# Twelve-channel LAN wavelength-division multiplexer on lithium niobate

**DOI:** 10.1515/nanoph-2023-0665

**Published:** 2023-12-25

**Authors:** Jianghao He, Ming Zhang, Dajian Liu, Yaoxin Bao, Chenlei Li, Bingcheng Pan, Yishu Huang, Zejie Yu, Liu Liu, Yaocheng Shi, Daoxin Dai

**Affiliations:** State Key Laboratory of Extreme Photonics and Instrumentation, Center for Optical and Electromagnetic Research, College of Optical Science and Engineering, International Research Center for Advanced Photonics, Ningbo Innovation Center, Zhejiang University, Hangzhou 310058, China; ZJU-Hangzhou Global Scientific and Technological Innovation Center, Zhejiang University, Hangzhou 311215, China; Jiaxing Key Laboratory of Photonic Sensing & Intelligent Imaging, Intelligent Optics & Photonics Research Center, Jiaxing Research Institute, Zhejiang University, Jiaxing 314000, China

**Keywords:** lithium-niobate-on-insulator, wavelength-division multiplexing, twelve single-channel bandpass filters, multimode waveguide gratings

## Abstract

A twelve-channel local-area-network (LAN) wavelength-division multiplexing (LWDM) filter is proposed and demonstrated with a uniform channel spacing of 4.5 nm (800 GHz) in the O-band of 1270–1330 nm by using x-cut lithium-niobate-on-insulator (LNOI) photonic waveguides for the first time. The present LWDM filter consists of twelve single-channel bandpass filters based on multimode waveguide gratings (MWGs) assisted with a TE_0_/TE_1_ mode (de)multiplexer. In particular, two stages of MWGs in cascade are introduced for each single-channel bandpass filter, in order to achieve high sidelobe suppression ratios, thus reducing interchannel crosstalk. For the fabricated twelve-channel LWDM filter, all the channels have very excellent box-like spectral responses with low excess losses of ∼0.6 dB, broad 1-dB bandwidths of ∼2.9–3.4 nm (which is close to 75 % of the channel spacing), and ultra-low interchannel crosstalk of <−40 dB in experiments. In addition, the present device is highly tolerant to the random variations of the etching depth (±20 nm) and the grating waveguide width (±20 nm) of the LNOI photonic waveguides, showing great potential for high-capacity WDM systems.

## 1 Introduction

Over the past few years, lithium-niobate-on-insulator (LNOI) has been developed and attracted intensive attention because of the high refractive-index contrast enabling optical waveguides with strong optical confinement, the wide transparency window range from 400 nm to 5000 nm enabling low-loss light propagation [1], [2], as well as the high second-order nonlinearity coefficient enabling electro-optic (EO) modulation [3], [4], [5], [6], [7], [8], [9], [10]. Currently, high-speed LNOI EO modulators have been realized with high-speed modulation up to 100 Gbps for a signal channel [3], [10] and have shown great potential for future high-capacity applications like wavelength-division multiplexing (WDM) systems. As it is well known, WDM is an attractive technology for significantly expanding the data communication capacity by incorporating multiple wavelength channels in a single-mode optical fiber [11]. WDM is categorized into dense WDM (DWDM) with a narrow channel spacing (e.g., 0.4, 0.8, or 1.6 nm) and coarse WDM (CWDM) with a large channel spacing (e.g., 20 nm). Recently, local-area network (LAN) WDM technology with a channel spacing of 800 GHz (∼4.5 nm) in the O-band has become attractive for interdatacenter applications due to its high spectral efficiency, high capacity, and long transmission distance [12], [13]. As a key functional component in WDM transceivers, high-performance optical filters have been playing an important role and numerous structures have been developed on various photonic integrated circuits, including micro-ring resonators (MRRs) [14], [15], [16], [17], arrayed-waveguide gratings (AWGs) [18], multimode interferometers (MMI) [19], cascaded Mach−Zehnder interferometers (MZIs) [20], [21], [22], and Bragg gratings [23], [24], [25], [26], [27], [28], etc. However, for x-cut LNOI waveguides considered in this paper as the most popular option for optical modulation, there might be non-negligible polarization crosstalk related to the mode hybridness as the light passes through the waveguide bends, which makes it difficult to implement compact and high-performance passive photonic filters with bends on x-cut LNOI, such as MRRs [14], [15], [16], [17] and AWGs [18].

Recently, great efforts have also been made to develop high-performance passive LNOI photonic devices, especially for WDM filters. In Ref. [29], a four-channel CWDM device based on SiN/LNOI MWGs was realized with a channel spacing of 20 nm. A DWDM filter based on grating-assisted contradirectional couplers (GACDC) was demonstrated by using hybrid SiN/LNOI photonic waveguides [30]. Furthermore, four-channel CWDM filters have also been realized with angled multimode interferometer (AMMI) [31] and multistage MZIs [32]. Among them, the fabrication processes in Refs. [29], [30] are impeding for large-scale LNOI photonic-integrated circuits. The AMMI-based CWDM filter [31] has a large footprint and lacks a flat-top response, while the MZI-based CWDM filter [32] needs to be designed and fabricated carefully. We have proposed a three-channel photonic filter based on MWGs on the LNOI platform for 50G passive optical networks with the channels of 1270/1300/1342 nm [33]. Nevertheless, WDM systems commonly require photonic filters, which feature narrower channel spacing and more wavelength channels to expand the communication capacity and to minimize the costs. For instance, the LAN WDM scheme with a uniform 4.5-nm channel spacing could serve as an attractive framework for handling 400G and 800G data rates [34]. Indeed, this places stringent requirements on WDM filters, such as negligible excess losses, ultra-low crosstalk, and channel uniformity.

In this paper, a novel twelve-channel LWDM filter with box-like spectral response is proposed and demonstrated with LNOI photonic waveguides for the first time. The present device is composed of twelve single-channel bandpass filters based on an apodized multimode waveguide gratings (MWGs) assisted with a two-mode (de)multiplexer. Each single-channel bandpass filter consists of two identical MWGs cascaded at the drop port for achieving high sidelobe suppression ratios and lowering the interchannel crosstalk. In experiments, all channels of the LWDM filter exhibit box-like spectral responses with low excess losses of ∼0.6 dB, broad 1-dB bandwidths of ∼2.9–3.4 nm (which is as large as ∼75 % of the channel spacing), and ultra-low interchannel crosstalk of <−40 dB. Additionally, both theoretical and experimental results indicate that the present device is highly tolerant to the random variations of the etching depth (±20 nm) and the grating width (±20 nm) of the LNOI photonic waveguides. Furthermore, the proposed device can be easily monolithic integrated with high-speed optical modulators to achieve ultra-large transmission capacity in optical links (like 12 × 200 Gb/s) in a single-mode waveguide.

## 2 Structure and design

**Figure 1:**
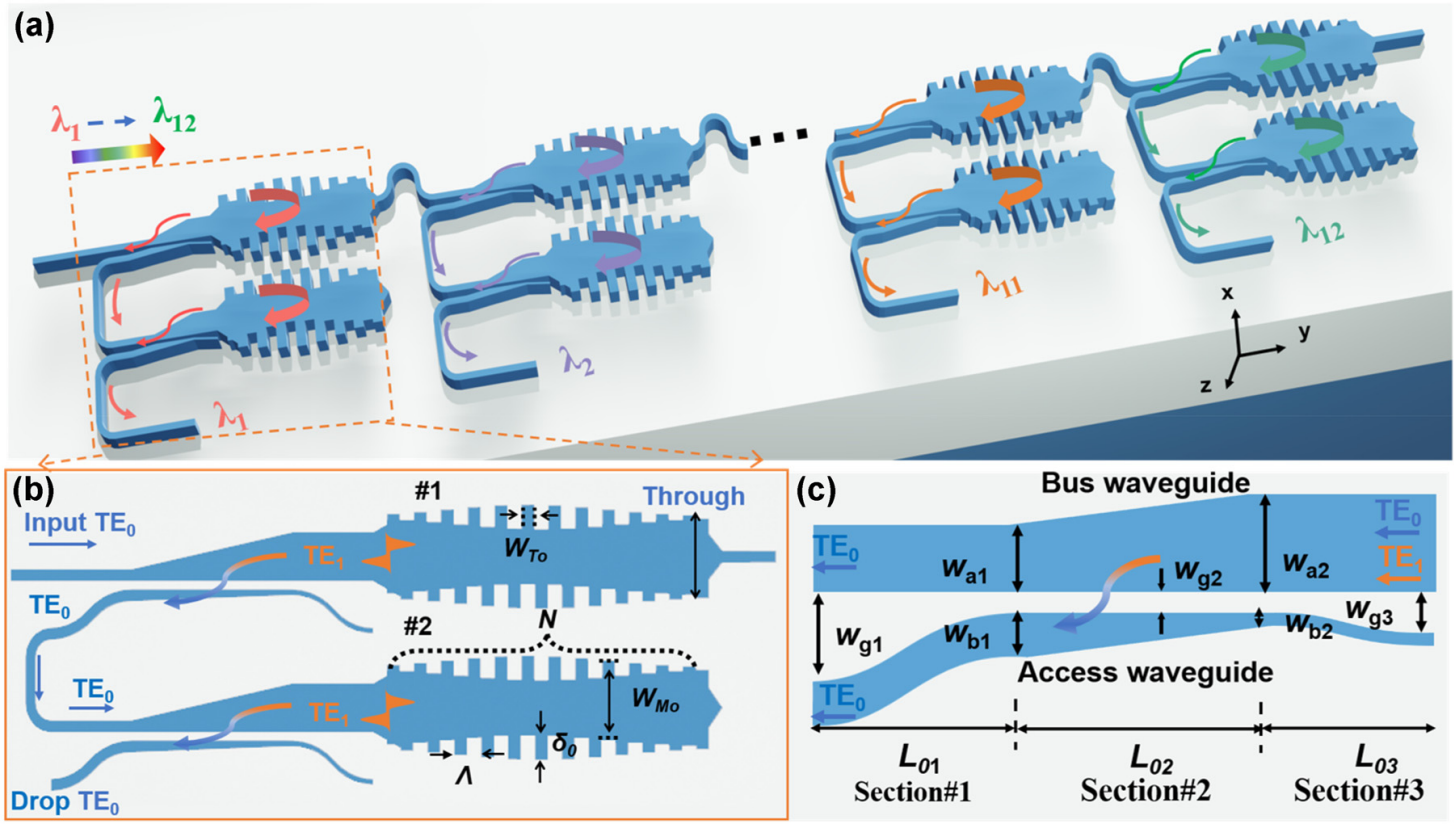
Schematic configurations. (a) The proposed twelve-channel LWDM filter on x-cut LNOI; (b) the single-channel bandpass filter realized with two identical MWGs in cascade; (c) the mode (de)multiplexer based on an adiabatic dual-core taper.

Figure 1(a) illustrates the proposed 12-channel LWDM filter, which consists of twelve single-channel bandpass filters based on (MWGs) assisted with the TE_0_/TE_1_ mode (de)multiplexers. In particular, for each single-channel bandpass filter, there are two identical cells (#1 and #2) in cascade to achieve high sidelobe suppression ratios and ultra-low interchannel crosstalk, as shown in Figure 1(b). Each cell integrates MWGs and a TE_0_/TE_1_ mode (de)multiplexer, as proposed in our previous work [28], [35]. Here, the dual-core taper structure is used for the TE_0_/TE_1_ mode (de)multiplexer, as shown in Figure 1(c), enabling low excess losses and low intermode crosstalk in a broad bandwidth. The MWGs is apodized and designed with antisymmetric corrugations on both sidewalls to facilitate contradirectional coupling of the forward TE_0_ mode and the backward TE_1_ mode at the Bragg wavelength band [36]. The backward TE_1_ mode is then converted to the TE_0_ mode and outputs from the drop port by using the TE_0_/TE_1_ mode (de)multiplexer. Meanwhile, the other wavelength channels pass through the MWGs of cell #1 lossless almost. With this design, it is possible to achieve box-like spectral responses with low losses and ultra-high sidelobe suppression at the drop port. In this paper, we consider air-cladded LNOI photonic waveguides with a 400-nm-thick x-cut thin-film LN and an etching depth (*H*_e_) of 200 nm as an example. Additionally, a bent waveguide is introduced between any two adjacent channels to reduce the higher order mode crosstalk and the undesired Fabry–Perot resonance.

In this design, the three-dimensional finite-difference time-domain (3D-FDTD) method with the boundary condition of perfectly matched layer (PML) is used to simulate the light propagation in our structures. The mode (de)multiplexers working for a broad wavelength band of 1250–1350 nm are realized by using adiabatic couplers [37], which intrinsically has a large fabrication tolerance because it works with the scheme of mode evolution. As shown in Figure 1(c), the mode (de)multiplexer consists of three parts, i.e., sections #1, #2, and #3. Sections #3 and #1 are used to separate the bus waveguide and the access single mode, while section #2 works as the major part of the mode conversion. The special design is that the width of the access waveguide is broadened down from *w*_b2_ to *w*_b1_, while the width of the bus waveguide is narrowed from *w*_a2_ to *w*_a1_ oppositely. Thus, the mode evolution between the TE_1_ and TE_0_ modes happens adiabatically by choosing a sufficiently long coupling region. In details, the core widths at the output/input ends of the bus and access waveguides for the adiabatic couplers are chosen as (*w*_a1_, *w*_a2_) = (1.3, 1.7) μm and (*w*_b1_, *w*_b2_) = (0.75, 0.2) μm, while the taper lengths are chosen as (*L*_01_, *L*_12_, *L*_23_) = (90, 100, 60) μm and the gap widths are chosen as (*w*_g1_, *w*_g2_, *w*_g3_) = (2, 0.25, 2) μm.

**Figure 2:**
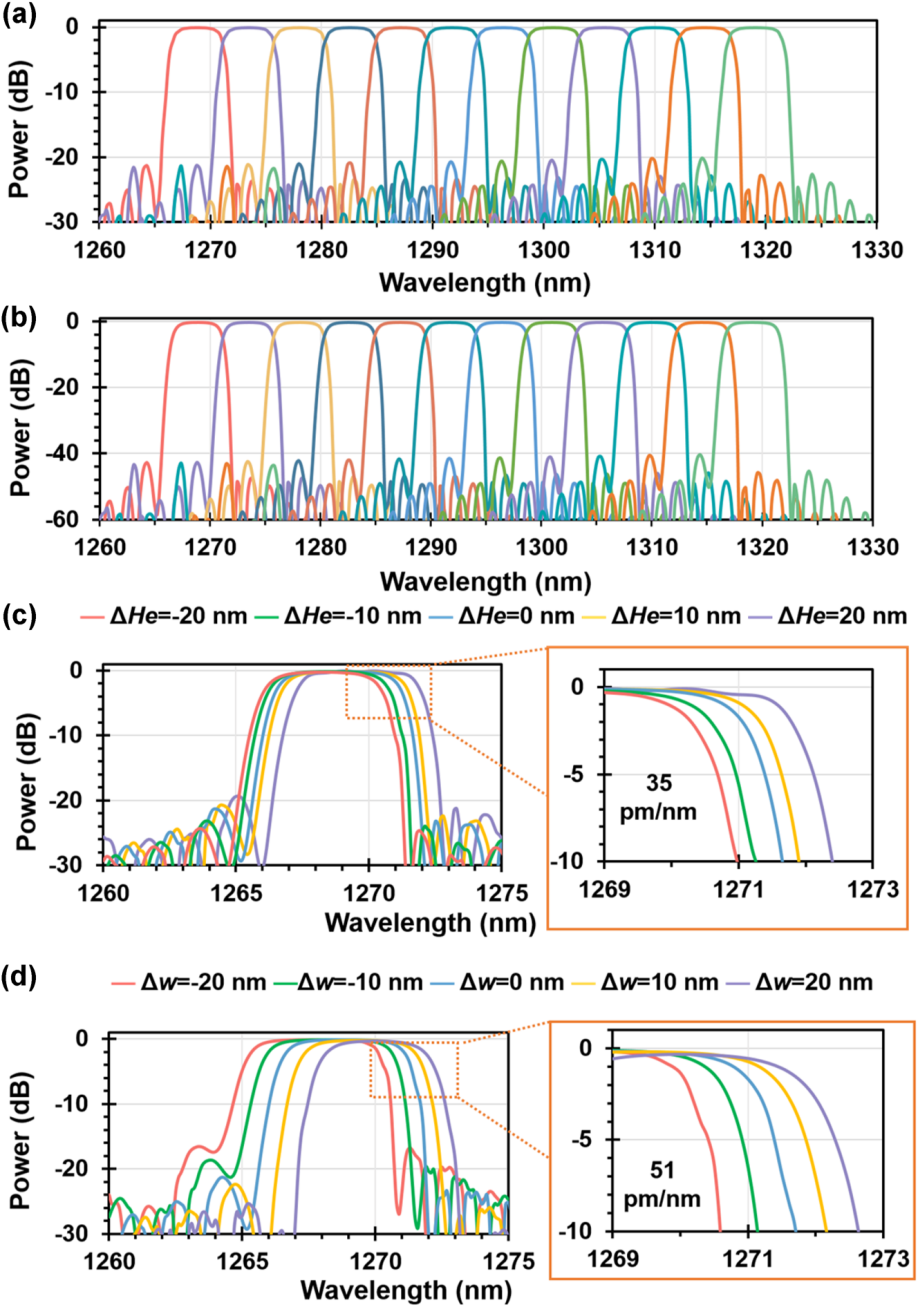
Simulation results of the proposed twelve-channel LWDM filter for single-channel bandpass filter with one cell (a) and two cells in cascade (b). Here, the grating period Λ linearly increases from 340 nm to 356.5 nm with a step of 1.5 nm. The parameters are *δ* = 300 nm, *W*_M0_ = 2 μm, *b* = 4, and *N* = 650. Simulated transmissions at the drop port of the designed MWGs filter when assuming there are some variations of the etching depth (c) and the grating waveguide width (d).

For the grating structure with a grating period Λ, there is a shifting of Λ/2 between the grating teeth at both sides to be asymmetric for realizing the conversion of the forward TE_0_ mode to the backward TE_1_ mode at the Bragg wavelength *λ*_B_. One has [26]

(1) $$\lambda_{B}=\left(n_{\text{eff0}}+n_{\text{eff}1}\right)\Lambda$$

where *n*_eff0_ and *n*_eff1_ are the effective indices of the forward propagating TE_0_ mode and the backward propagating TE_1_ mode in the MWGs, respectively, *λ*_B_ is the expected resonant Bragg wavelength. It is known that the reflection *R* and the bandwidth Δ*λ* of the structure are related to the grating geometry and the grating length *L*, i.e., [38]

(2) $$R=\tanh^{2}\left(kL\right),$$

(3) $$\Delta \lambda =\frac{\lambda^{2}}{n_{\text{g}}L}\sqrt{1+{\left(\frac{kL}{\pi }\right)}^{2}},$$

where *n*_g_ is the group index and *κ* is the coupling coefficient. To obtain an appropriate bandwidth Δ*λ* of ∼3 nm for the MWGs-based optical filters, one can simultaneously weaken the coupling coefficient and increase the coupling length according to Eqs. (2) and (3). However, to achieve a weak coupling coefficient, one has to reduce the corrugation depth *δ*_0_ [30] and thus a high fabrication resolution is often demanded. Alternatively, another option is to increase the grating waveguide width *W*_M0_ and maintain a large corrugation depth *δ*_0_, in which case, however, higher-order mode hybridization might be introduced [39]. Thus, the corrugation depth *δ*_0_ and the grating waveguide width *W*_M0_ of MWGs should be chosen by making a trade-off regarding the mode hybridization and the fabrication ability. Here, the corrugation depth *δ*_0_ is designed up to 300 nm, the grating waveguide width *W*_M0_ is set to 2 μm, and the grating period *N* is set to 650 to ensure a narrow-band photonic filter with low losses. Furthermore, to achieve a high sidelobe-suppression ratio (SLSR) and reduce the interchannel crosstalk, a Gaussian-profile apodization is introduced to taper the coupling strength along the MWGs. The multimode waveguide width *W*_M0_ of the MWGs is fixed, and the corrugation is achieved by offsetting the core region from the central axis by ±*δ*/2 as shown in Figure 1(b) and more detail about the design can be found in our previous work [35]. As shown in Figure 1(b), the offset *δ* is defined as

(4) $$\delta =\delta_{0}\exp\left[-b{\left(i/N-1/2\right)}^{2}\right]$$

where *δ*_0_ is the maximum corrugation depth, *b* is the apodization index, *i* = 1, …, *N*, and *N* is the total period number of the grating period. With this design, the SLSR is ∼20 dB when the apodization index *b* is set to 4, as shown in Figure 2(a). Note that the SLSR can be further improved to 40 dB by cascading two identical cells (i.e., #1 and #2) for each single-channel bandpass filter, as shown in Figure 2(a) and (b). Finally, the photonic filters working for the other channel wavelengths can be realized by appropriately modifying the grating period Λ according to the phase matching [26] while the other parameters of the MWGs are kept unchanged. For the presented 12-channel LWDM filter, the grating period Λ linearly increases from 340 nm to 356.5 nm in steps of 1.5 nm. The presented twelve-channel LWDM filter features a box-like response with a 1-dB bandwidth of ∼3.5 nm, low excess losses of <0.2 dB, and ultra-low crosstalk of <−40 dB, as shown in Figure 2(b).

In experiments, it is quite challenging to precisely control the lateral dimension and the etch depth *H*_e_ for the present waveguide structures [40]. Figure 2(c) shows the calculated spectral responses of the MWGs when assuming that the etching-depth variation is given as Δ*H*_e_ = 0, ±10, and ±20 nm. It can be seen that the center wavelength has a small redshift of ∼1.4 nm when the etching depth varies from 180 nm to 220 nm. Note that the 1-dB bandwidth and the SLSR remain the same almost even when Δ*H*_e_ *=* ±20 nm. Figure 2(d) shows the calculated spectral responses when assuming the grating structure has a lateral-dimension variation of Δ*w* = 0, ±10, and ±20 nm. Accordingly, the width of grating teeth is given *W*_T_ = *W*_T0_ + Δ*w*, and the width of the multimode waveguide is given by *W*_M_ = *W*_M0_ + Δ*w* (where *W*_T0_ and *W*_M0_ are the design values). From Figure 2(c), it can be seen that the excess loss and the 1-dB bandwidth are kept unchanged almost, while the center wavelength changes linearly with a slope of 51 pm/nm. In summary, the present LNOI photonic filter is highly tolerant to the random variations of the etching depth and the waveguide width, despite that the central wavelength has some shift due to the dimensional variation as expected.

## 3 Fabrication and measurement

**Figure 3:**
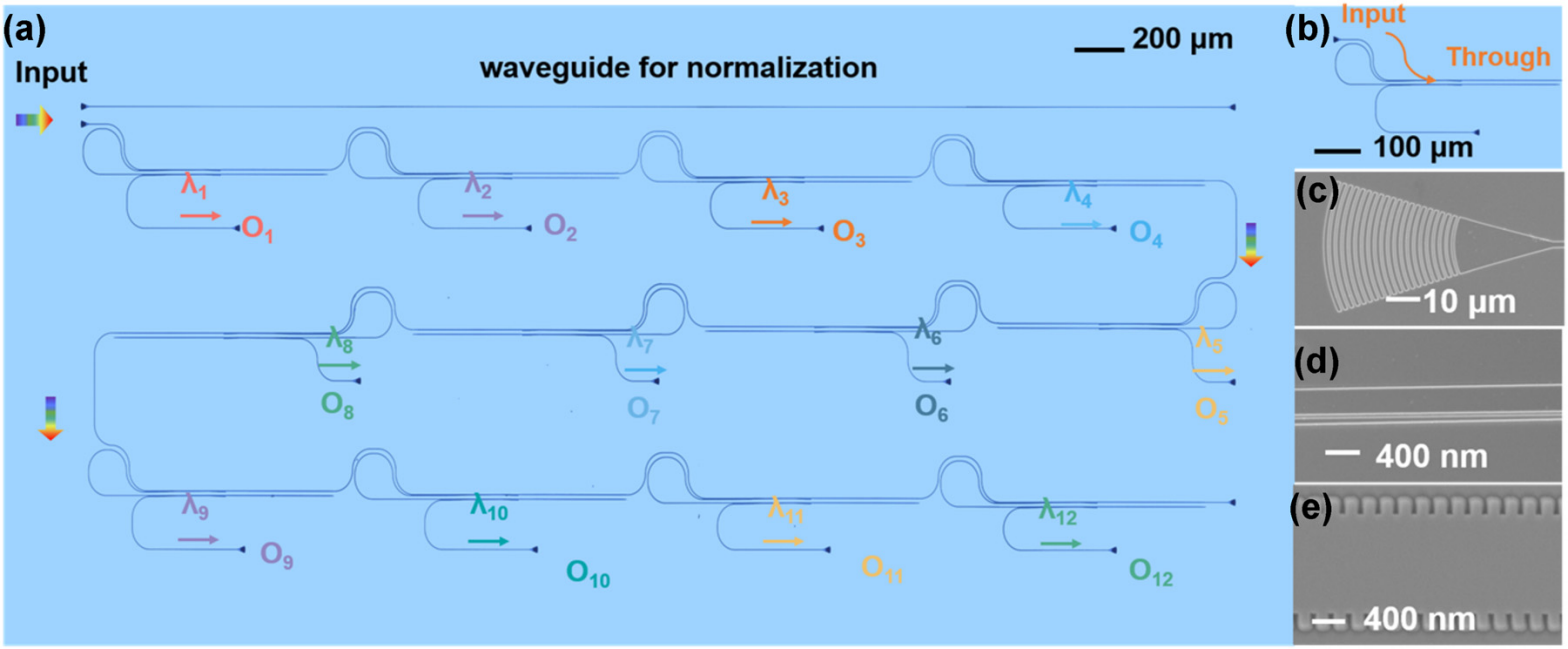
Optical microscope image of the fabricated device. (a) The 12-channel LWDM filter; (b) the single-channel bandpass filter with two identical cells in cascade. SEM images of the grating coupler (c), the mode (de)multiplexer (d), and the MWGs (e).

The designed 12-channel LWDM filter was fabricated using electron beam lithography (EBL) to pattern the device structure, and it was etched by 200 nm with the Ar+ plasma dry-etching process. The chip has a 400-nm-thick top LN layer, a 3-μm buried silica layer, and a 500 μm silicon substrate. Grating couplers for TE polarization were employed for efficient fiber-chip interconnects, and straight single-mode waveguides were also fabricated on the same chip as the reference. The optical microscope and scanning electron microscope (SEM) images of the fabricated 12-channel LWDM filter are shown in Figure 3(a)–(e).

**Figure 4:**
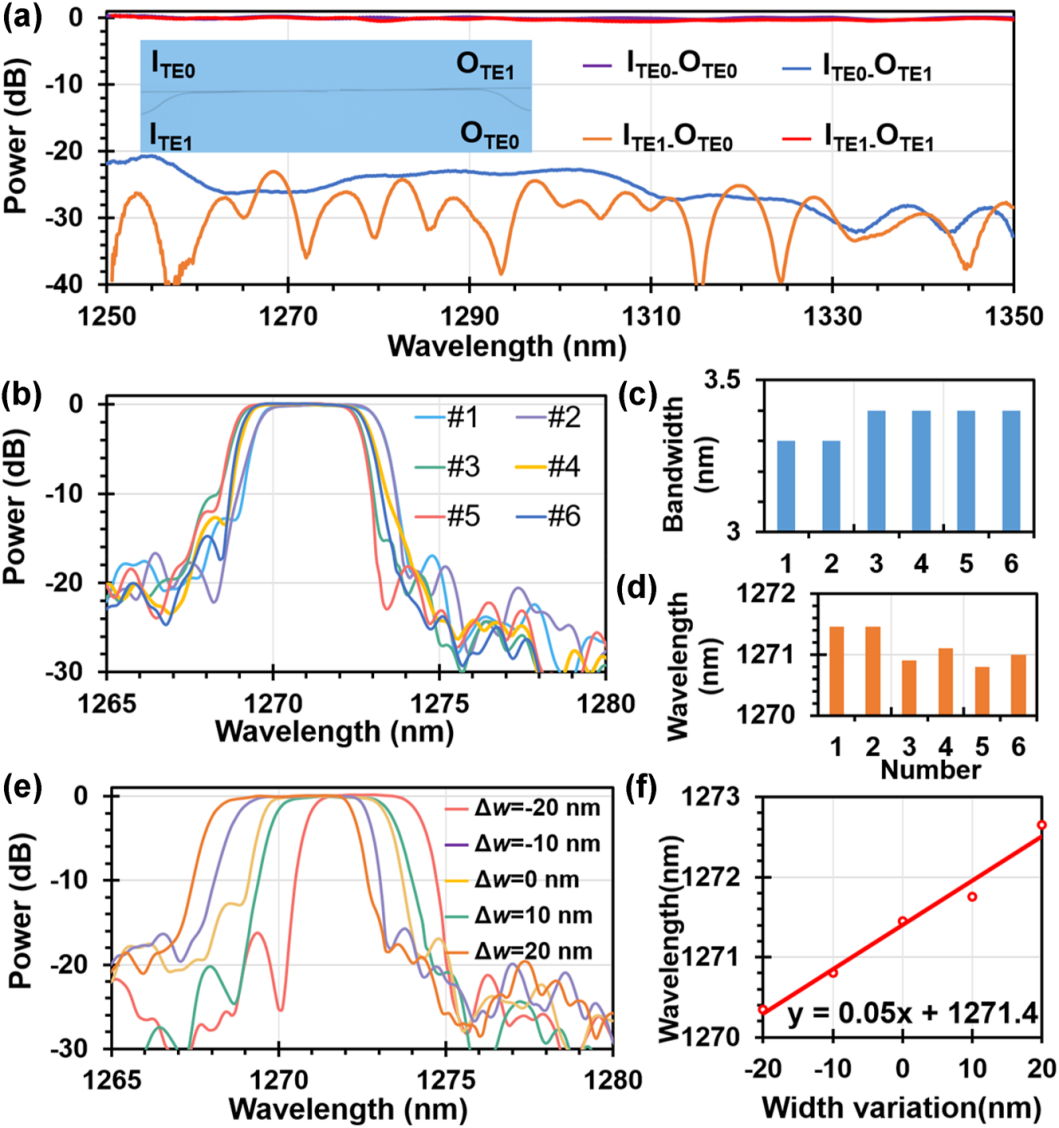
Measurement results of the fabricated device. (a) Measured transmissions from the input port (I_TE0,_ I_TE1_) to the output port (O_TE0_, O_TE1_) for the fabricated photonic integrated circuit composed of a pair of mode (de)multiplexers; (b) measured spectral responses at the drop port of six identical MWGs photonic filters fabricated on the same chip, and the corresponding 3-dB bandwidth (c) as well as the measured center-wavelength (d); (e) measured transmissions spectra of the MWGs photonic filters as the grating waveguide width has a variation of Δ*w* = −20, 0,+20 nm; (f) measured central wavelength shift for the cases with different grating waveguide width variation.

The fabricated device was characterized using a broadband super-luminescence light emitting diode (SLED) at the O-band (Thorlabs S5FC1021P), a polarization controller (PC), and an optical spectrum analyzer (OSA). The continuous-wave broadband light from the SLED was transmitted through the PC first and the TE-polarized light was then coupled into and out of the chip through the grating couplers. Finally, the transmissions were characterized by using an OSA. Figure 4(a) shows the measured transmissions from the input port (I_TE0,_ I_TE1_) to the output port (O_TE0_, O_TE1_) for the fabricated photonic-integrated circuit composed of a pair of mode (de)multiplexers, as shown by the inset in Figure 4(a). The fabricated mode (de)multiplexer has an ultra-low excess loss of 0.1 dB and low crosstalk of less than −20 dB for these two modes in a broad wavelength range of 1250–1350 nm, which covers the wide operation wavelength window of our twelve-channel LWDM filter.

To evaluate the uniformity of the fabricated MWGs photonic filters, there are six devices with identical designs fabricated on the same chip. Figure 4(b) shows the measurement results for these six samples, which exhibit excellent spectral responses with well-aligned central wavelengths around 1271 nm, similar 1-dB bandwidths of ∼3.4 nm, and a high SLSR about 17–20 dB. Figure 4(c) and (d) give more details about the statistical results for the central wavelengths and 1-dB bandwidths of these six samples. It can be seen that the mean deviations for the central wavelength and the bandwidth are ∼0.4 nm and ∼0.1 nm, respectively, indicating that the present MWGs photonic filter on LNOI is very promising for LWDM applications.

To verify the fabrication tolerance of the devices, the MWGs photonic filters with different grating waveguide widths of Δ*w* = 0, ±10, and ±20 nm were also fabricated on the same chip and the measured results are shown in Figure 4(e). From this figure, one sees that the present photonic filters still have a box-like spectral response with low excess losses of <0.1 dB and a high SLSR up to 15 dB even when the grating waveguide width varies with Δ*w* = ±20 nm. As illustrated in Figure 4(f), the center wavelength experiences a linear red red-shift due to the increasing effective indices for both TE_0_ and TE_1_ modes, which agrees well with the simulation results obtained in Figure 2(c) and proves the presented MWGs-based filters have a relatively large fabrication tolerance.

**Figure 5:**
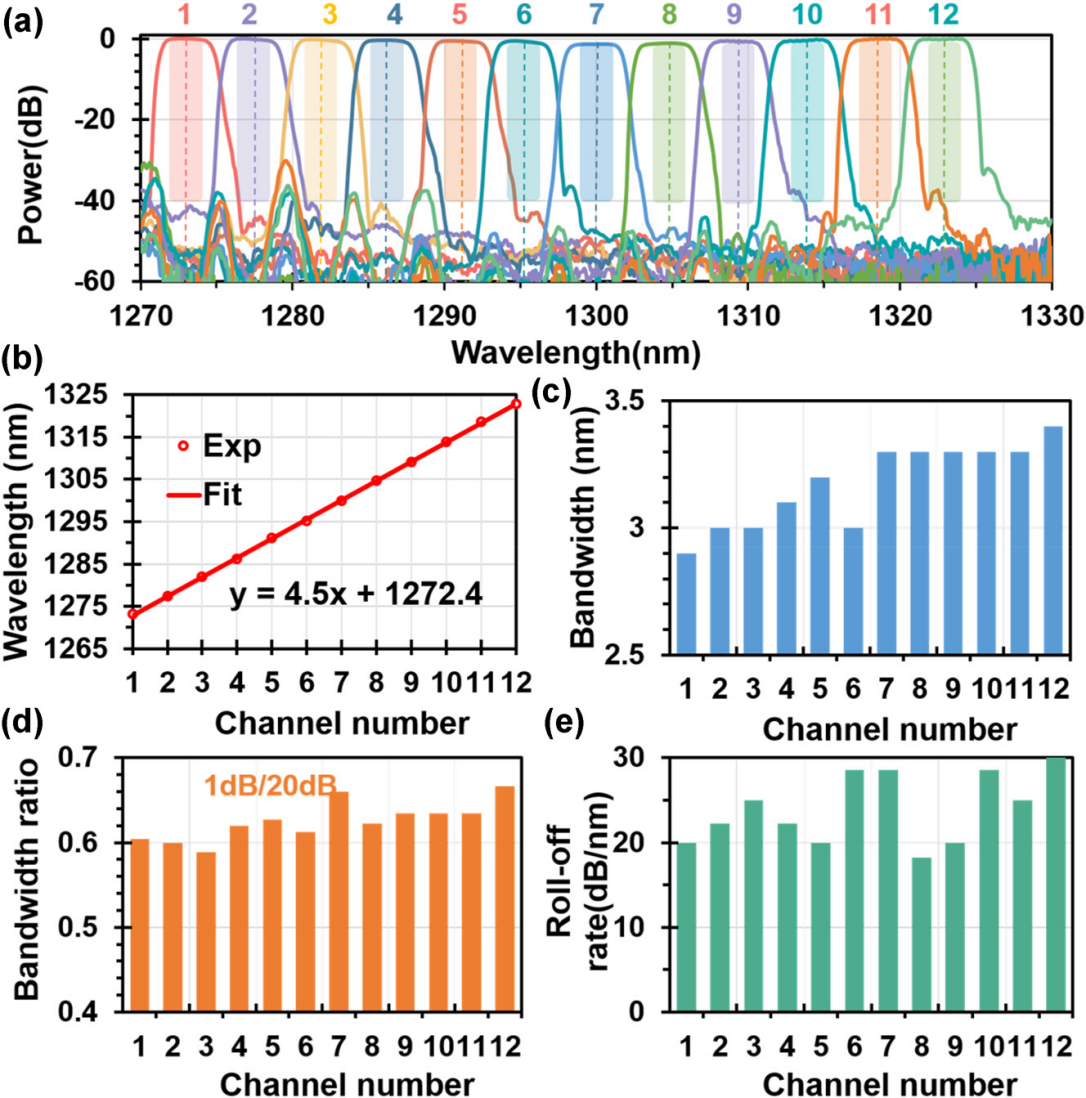
Measured results of the proposed twelve-channel LWDM filter. (a) Measured spectral responses at the drop port of the fabricated 12-channel LWDM filter; the center wavelength (b) and the 3-dB bandwidth (c) of all twelve channels.

The transmittance spectra of the fabricated 12-channel LWDM filter were measured at ports O_1_–O_12_, as shown in Figure 5(a). It should be noted that each single-channel bandpass filter consists of two identical cells cascaded at the drop port, as shown in Figure 3(b). With the design, the SLSR is significantly improved to 40 dB, which agrees very well with the theoretical results. It is possible to achieve higher SLSR by cascading more cells at the drop port. The adjacent-channel and nonadjacent-channel crosstalks are −40 to −50 dB and −45 to −55 dB at the center wavelengths for all the channels, respectively. For LWDM system with a channel spacing of 4.5 nm, it is usually required to check the excess loss and the interchannel crosstalk in the 2.2-nm bandwidth around the center wavelengths [as defined by the shallow regions in Figure 5(a)]. For the present 12-channel LWDM filter, the interchannel crosstalk in the 2.2-nm bandwidth is lower than −40 dB for most channels (which can be improved further by narrowing the bandwidth appropriately). All the channels have uniform spectral responses with low excess losses of ∼0.2–0.6 dB (excluding the coupling loss of grating couplers). Note that the 7th and 8th channels have excess losses up to ∼1.2 dB, which is due to the fabrication imperfections. The central wavelengths for all 12 channels are illustrated in Figure 5(b), showing excellent linearity with a channel spacing of approximately 4.5 nm. The measured LWDM filter has a box-like response with 1-dB bandwidths are 2.9–3.4 nm, a ratio of 1 dB/20 dB bandwidth as high as ∼0.59–0.67, and the roll-off rate is 18–30 dB/nm of all channels, as demonstrated in Figure 5(c)–(e). Table 1 presents a summary of the performance of the photonic filters on LNOI reported in recent years. It can be seen that the present work is remarkable regarding its low excess loss and ultra-low interchannel crosstalk and high channel number. It indicates that the proposed 12-channel LWDM filter works well with high fabrication tolerance and provides a promising option for realizing high capacity data transmissions when integrated with the array of high-speed EO modulators on the same chip [3].

**Table 1:** Comparison of x-cut LNOI photonic filters reported recently.

Refs	Structure	Excess loss (dB)	Channel spacing (nm)	Crosstalk at the center wavelength (dB)	Wavelength channel
[28]	Hybrid SIN-LNOI MWGs	∼1.5	20	−18	4
[29]	Hybrid SIN-LNOI GACDC	∼2.5	3.2	−20	3
[30]	AMMI	∼0.7	20	−18	4
[31]	MZIs	∼1.5	20	−8	4
[32]	MWGs	∼0.2	30/42	−30	3
This work	MWGs	**∼0.6**	**4.5**	**−40 to −50**	**12**

The bold values are to compare our better performance with the reference.

## 4 Conclusions

In conclusion, we have successfully designed and fabricated a 12-channel LWDM filter with low excess losses and ultra-low channel crosstalk on the x-cut LNOI platform. The grating waveguide width *W*_M0_ has been designed carefully to prevent the excitation of higher-order modes while the corrugation depth *δ*_0_ has been designed to achieve a narrow-bandwidth photonic filter. Additionally, we have introduced two cells of MWGs in cascade to improve the SLSR and reduce interchannel crosstalk significantly. The fabricated LWDM filter has shown a uniform channel spacing of ∼4.5 nm and a box-like spectral response with a 1-dB bandwidth of ∼2.9–3.5 nm (which is near 75 % of the channel spacing). The measured results have also demonstrated ultra-low excess losses of ∼0.2–0.6 dB and ultra-low interchannel crosstalk of <−40 dB for all channels (except the 7th and 8th channels due to the fabrication defects). Moreover, the proposed LNOI LWDM filter is tolerant to the random variations of the etching depth (±20 nm) and the grating waveguide width (±20 nm). This present 12-channel LWDM filter with high performance is expected to play a pivotal role in the development of advancing LNOI photonic-integrated circuits, paving the way for high-capacity data transmissions.
